# A phase I trial of LXS196, a protein kinase C (PKC) inhibitor, for metastatic uveal melanoma

**DOI:** 10.1038/s41416-022-02133-6

**Published:** 2023-01-09

**Authors:** S. Piperno-Neumann, M. S. Carlino, V. Boni, D. Loirat, F. M. Speetjens, J. J. Park, E. Calvo, R. D. Carvajal, M. Nyakas, J. Gonzalez-Maffe, X. Zhu, M. D. Shirley, T. Ramkumar, A. Fessehatsion, H. E. Burks, P. Yerramilli-Rao, E. Kapiteijn

**Affiliations:** 1 Institut Curie, Paris, France; 2 Blacktown and Westmead Hospitals, Sydney, NSW Australia; 3 Melanoma Institute Australia, The University of Sydney, Sydney, NSW Australia; 4 START Madrid-CIOCC, Centro Integral Oncológico Clara Campal, Madrid, Spain; 5 Leiden University Medical Center, Leiden, The Netherlands; 6 Columbia University Irving Medical Center, New York, NY USA; 7 Oslo University Hospital, Oslo, Norway; 8 Novartis Pharma AG, Basel, Switzerland; 9 Novartis Institutes for BioMedical Research, Cambridge, MA USA

**Keywords:** Cancer, Eye cancer

## Abstract

**Background:**

Up to 50% of patients with uveal melanoma develop metastases (MUM) with a poor prognosis and median overall survival of approximately 1 year.

**Methods:**

This phase I study evaluated the safety, tolerability, pharmacokinetics, pharmacodynamics and efficacy of the oral protein kinase C inhibitor LXS196 in 68 patients with MUM (NCT02601378). Patients received LXS196 doses ranging from 100–1000 mg once daily (QD; *n* = 38) and 200–400 mg twice daily (BID; *n* = 30).

**Results:**

First cycle dose-limiting toxicities (DLTs) were observed in 7/38 (18.4%) QD and 2/17 (11.8%) BID patients. Hypotension was the most common DLT, occurring at doses ≥500 mg/day, and manageable with LXS196 interruption and dose reduction. Median duration of exposure to LXS196 was 3.71 months (range: 1.81–15.28) for QD and 4.6 months (range: 0.33–58.32) for BID dosing. Clinical activity was observed in 6/66 (9.1%) evaluable patients achieving response (CR/PR), with a median duration of response of 10.15 months (range: 2.99–41.95); 45/66 had stable disease (SD) per RECIST v1.1. At 300 mg BID, the recommended dose for expansion, 2/18 (11.1%) evaluable patients achieved PR and 12/18 (66.7%) had SD.

**Conclusion:**

These results suggest manageable toxicity and encouraging clinical activity of single-agent LXS196 in patients with MUM.

## Background

Uveal melanoma (UM) is the most common primary intraocular malignant tumour in adults, involving the iris, ciliary body or choroid, and is biologically distinct from cutaneous melanoma in terms of driver genes and mutational burden [1]. A meta-analysis of 22 studies published from 1943 to 2015 demonstrated an incidence rate of 5.74 (95% confidence interval [CI]: 4.37–7.11) and 7.30 (95% CI: 6.36–8.24) in the USA and Europe, respectively [2]. Nearly 50% of patients with UM develop metastatic disease within 15 years of their initial diagnosis. Frequent sites of metastasis are liver (95%), lungs (24%), bone (16%) and skin (11%) [3]. A recent meta-analysis in 912 patients with metastatic uveal melanoma (MUM) enroled in prospective studies showed a median overall survival (OS) of 10.2 months and a 1-year OS rate of 43%. For patients who received treatment with an anti-neoplastic agent, the median OS was 9.3 months, and the 1-year OS rate was 38.4%; median progression-free survival (PFS) was 2.8 months, and the 6-month PFS rate was 21.5% [4]. MUM is refractory to chemotherapy [5] and anti-PD-1 therapies have shown limited activity, possibly due to the low mutational burden of this rare melanoma [6]. Combinations of anti-CTLA4 and anti-PD-1 immunotherapies in two recent single-arm phase II trials showed a median PFS of 3.0 and 5.5 months, and a median OS of 12.7 and 19.1 months, respectively, after a short follow-up of 13 months [7, 8]. Most recently, a randomised phase III trial with a bispecific fusion protein (tebentafusp, Immunocore), designed to redirect T cells to gp100-positive cells compared to Investigator’s choice (dacarbazine, ipilimumab, or pembrolizumab) in HLA-A*02:01-positive patients with first-line MUM, demonstrated a 1-year OS rate of 73% in the tebentafusp group and 59% in the control group (hazard ratio: 0.51; 95% CI: 0.37–0.71). However, only a minor improvement in PFS and no improvement in overall response rate (ORR) was demonstrated [9]. Based on the mechanism of action, a limitation in the therapeutic approach of tebentafusp is the requirement for patients to be HLA-A*02:01-positive, thereby excluding around 50% of patients who have no current systemic treatment for metastatic disease, which has been proven to improve OS. Outcomes for patients with metastatic disease who are not eligible for or who are refractory to tebentafusp remain extremely poor.

Somatic mutations affecting either one of two genes, guanine nucleotide-binding protein alpha-Q (GNAQ) or guanine nucleotide-binding protein alpha-11 (GNA11), that encode G-protein alpha subunits of heterotrimeric G-protein-coupled receptor (GPCR) complexes have been identified in >90% of patients with MUM [10–12]. In *GNAQ* and *GNA11* wildtype MUMs, mutations in other G-protein pathway-associated genes, CYSTLR2 (4%) and PLCB4 (2.5%), have also been identified [13]. GPCRs are increasingly recognised as promoters of malignancy in diverse cell types, including melanocytes [14]. The incidence of mutations affecting *GNA11* or *GNAQ* in melanocytic neoplasms varies with clinical setting, suggesting that the products of the two genes contribute unequally to the biology of UM [11]. A key downstream player of the constitutively active G-protein alpha subunits (*GNAQ* or *GNA11*) is the phospholipase C/protein kinase C (PKC) signalling pathway. PKC signalling is a key node in the maintenance of cellular homoeostasis; in UM cells, it results in phosphorylation and increased transcript expression of RasGrp3, a guanine nucleotide exchange factor that transduces signalling from *GNAQ/GNA11* to the MAPK signalling pathway through PKC delta [15]. Preclinical data showed selective sensitivity of UM cell lines carrying Gα subunit mutations when cell lines were treated with PKC inhibitors, including AEB071 [16]. Thus, PKC inhibitors may be considered a viable treatment option for MUM. AEB071 (also known as sotrastaurin), a first-generation, oral, pan-PKC inhibitor of both the classical (α, β) and novel (δ, ε, η, θ) forms of PKC [17], was tested in a phase I dose-escalation study in patients with MUM. Modest clinical activity was demonstrated with stable disease (SD) as the best response in 50% of patients treated, and increased frequency of dose-limiting gastrointestinal (GI) toxicities associated with increasing dose [12].

LXS196 is a potent, second-generation, oral PKC inhibitor designed with improved pharmaceutical properties compared with AEB071. LXS196 has a highly selective kinase profile affording increased tolerability in preclinical studies, with cellular activity restricted to UM cell lines containing mutant *GNAQ* or *GNA11* and no activity observed in skin-derived melanoma cell lines driven by mutant B-Raf or N-Ras [18]. When assessed in the 92.1 human UM mouse xenograft model, LXS196 dosed as a single agent leads to tumour regression at doses below its maximum tolerated dose (MTD), in contrast to AEB071, which achieves only stasis in this model [19]. Data from non-clinical single-dose and 4-week repeated dose toxicology studies in dog, suggested that a decrease in systolic blood pressure may be a potential toxicity observed in patients treated with LXS196. The 4-week toxicology study also identified the GI tract as a potential target of LXS196. All findings demonstrated partial to complete reversibility during the 4-week recovery phase and were deemed readily monitorable in clinical settings [20].

In this phase I trial, we aimed to evaluate the safety, preliminary efficacy, pharmacokinetics (PK) and pharmacodynamics (PD) of LXS196, and to determine the MTD and/or recommended dose for expansion (RDE) of LXS196 as a single agent in patients with MUM. Retrospective genomic analyses were conducted on baseline metastatic tumour biopsies.

## Materials and methods

This was a phase I, first-in human, multicentre, open-label study (NCT02601378), designed and sponsored by Novartis Pharmaceuticals Corporation and initiated on 1 February 2016. The study protocol and amendments were approved by the Independent Ethics Committee or Institutional Review Board for each centre, and all patients provided written informed consent. The study was conducted according to the principles of the Declaration of Helsinki and performed in compliance with Good Clinical Practice. All patients were aged ≥18 years old and had biopsy-proven MUM with progressive and measurable disease. Patients were either treatment naïve or their disease had progressed (radiologically or clinically) on their most recent therapy. There was no eligibility limit to the number of prior lines of therapy, including PKC inhibitors other than LXS196. Patients were required to have an Eastern Cooperative Oncology Group (ECOG) performance status of ≤1 and requested to provide a tumour biopsy at baseline (pre-treatment) and on treatment at day 15 of cycle 1 (C1D15). Exclusion criteria included impaired cardiac function or clinically significant cardiac diseases, receipt of concomitant medications known to be strong inducers or inhibitors of cytochrome P450 3A4/5 or known for QT prolongation risk, and impaired GI function that could interfere with absorption of LXS196.

The primary objectives were to characterise the safety and tolerability and identify the MTD and/or RDE of LXS196 as a single agent in patients with MUM. Secondary objectives included investigation of the preliminary antitumour activity of LXS196 and evaluation of the PK and PD of LXS196. Exploratory objectives included assessment of mutations in cancer driver genes by transcriptome and targeted DNA sequencing of baseline tumour biopsies.

During dose escalation, patients received oral LXS196 either once daily (QD; 100–1000 mg) or twice daily (BID; 200–400 mg) in 28-day cycles until disease progression, intolerable toxicity or withdrawal of consent (Supplementary Fig. 1). Dosing was omitted on the second day of the first cycle (C1D2) to allow for longer post-dose sampling in order to better characterise PK. LXS196 was given on an empty stomach, at least 1 h before or 2 h after a meal. Dose escalation was guided by safety, PK data and a two-parameter Bayesian Logistic Regression Model (BLRM) [21] employing escalation with overdose control (EWOC) criteria [22]. The DLT period lasted 28 days after the first dose of LXS196. A patient was evaluable for the BLRM if the patient took at least 75% of the planned doses during the DLT period or had a DLT. Upon determination of the MTD/RDE, the expansion part of the study was opened to further evaluate the safety, tolerability, PK and PD of LXS196 at the RDE in patients with MUM. No hypothesis testing was planned in this study, and therefore, no formal sample size calculation was needed. The sample size was selected to ensure the BLRM had adequate operating characteristics when selecting the MTD/RDE.

Routine safety assessments, including laboratory assessments, physical examinations, vital signs and electrocardiograms (ECGs), were conducted at regular intervals throughout the study and more frequently as needed following patient assessment by the treating physician. Adverse events (AEs) were assessed continuously according to the National Cancer Institute Common Terminology Criteria for Adverse Events (NCI-CTCAE) v4.03. For patients who did not tolerate their assigned dosing schedule due to a treatment-related AE, dose adjustments were permitted to allow the patient to continue study treatment once the AE had resolved to ≤grade 1.

Tumour response was assessed locally by computed tomography (CT) and/or magnetic resonance imaging (MRI) of the chest, abdomen and pelvis, according to Response Evaluation Criteria in Solid Tumours (RECIST) v1.1 at baseline, on C3D1 and every two cycles thereafter until cycle 11. After 11 cycles of treatment, response was evaluated every three cycles until the end of treatment.

In the dose escalation part of the study, PK samples were collected at various pre- and post-dose time points on C1D1, D2, D3, D15 and D16. Drug concentrations were assessed using a validated liquid chromatography-tandem mass spectrometry assay. PK parameters included area under the curve (AUC_0–12h_), maximum concentration (*C*_max_) and time to *C*_max_ (*T*_max_) on C1D1 and C1D15, and additionally, the accumulation ratio (*R*_acc_) of AUC_0–*t*_ on C1D15 compared to that on C1D1.

Fresh metastatic tumour biopsies were collected at baseline (prior to first dose) and on treatment at C1D15 to evaluate the modulation of PKC substrate proteins in tumours following exposure to LXS196 and to perform exploratory transcriptome and targeted DNA sequencing. Whole blood samples (peripheral blood mononuclear cells; PBMCs) were collected pre-dose on C1D1 and D15, and at various time points post-dose on C1D1, D2, D3, D15 and D16 to evaluate the modulation of PKC substrate proteins in a surrogate tissue. Owing to the exploratory nature of this phase I study, formal statistical tests were not planned to be performed. Data are presented using descriptive statistics and visualisations. Continuous variables are described with the mean and range. Categorical variables are described using counts and percentages. PFS is described using the Kaplan–Meier (KM) method.

### Pharmacodynamics

PD analysis for the proximal PD markers pPKC delta and phosphorylated myristoylated alanine-rich C-kinase substrate (MARCKS; pMARCKS) was performed in pre- and on-treatment tumour biopsies from patients who received LXS196 on a QD schedule. PD analysis was not performed on tumour samples from patients who received LXS196 on a BID schedule.

Custom assays were developed to measure the status of the PKC pathway. In brief, tumour tissue was homogenised in lysis buffer, and total protein concentrations were determined using a bicinchoninic acid (BCA) assay (Pierce). The samples were then run on the Meso Scale Discovery platform to measure total and pMARCKS. A seven-point calibration curve based on the 92.1 UM cell line was used to determine the concentration of total and pMARCKS in patient samples. All samples were run in duplicate wells. Total and phosphorylated PKCdelta (PKCd; pPKCd, phosphorylated at S299) levels were determined using microcapillary electrophoresis on the Sally Sue platform (Protein Simple, San Jose, CA, USA). Area under the peaks corresponding to the target was measured and used for further calculation. All results were normalised for the amount of protein used in each assay, and the ratio of phosphorylated protein to the corresponding total protein was reported. The calculation of data for analysis are described in the supplementary appendix.

### Sequencing data generation and analysis

Detailed DNA and RNA extraction methods and analysis are described in the supplementary appendix [23–35].

## Results

### Patient characteristics

A total of 68 patients with MUM were treated with LXS196 in this first-in-human study: 56 in the dose escalation part and 12 in dose expansion (Supplementary Fig. 1). Patients enroled in dose escalation received doses ranging from 100–1000 mg QD (*n* = 38) and 200–400 mg BID (*n* = 18) until the RDE was established. Patients enroled in the expansion part (*n* = 12) received LXS196 at the RDE. Supplementary Table 1 shows patient disposition. At the final data cut-off date (7 January 2022), all patients in the QD schedule had discontinued treatment due to disease progression. Patient demographics and baseline characteristics are shown in Table 1. The median age was 56.0 years, 36 (52.9%) were male, and most (*n* = 62; 91.2%) had a performance status of 0. The majority of patients (*n* = 60; 88.2%) had liver metastases, 27 of whom (14 in the BID schedule and 13 in the QD schedule) had only liver metastases and no other sites of disease at study entry. Other reported metastatic sites with an incidence of at least 10% included lung (n = 18, 26.5%), bone (*n* = 13, 19.1%), lymph node (*n* = 8, 11.8%) and skin (*n* = 7, 10.3%). Lactate dehydrogenase (LDH) levels noted to be above the upper limit of normal (ULN) at study entry were reported in 38 patients (15 in the BID schedule and 23 in the QD schedule).

**Table 1:** Summary of baseline patient characteristics.

Characteristic		All LXS196 QD patients *n* = 38	All LXS196 BID patients *n* = 30	LXS196 300 mg BID (RDE) patients *n* = 18	All LXS196 patients *N* = 68
Median age, years (range)		55 (26–76)	57 (33–78)	56.5 (38–76)	56 (26–78)
Sex, *n* (%)	Male	22 (57.9)	14 (46.7)	7 (38.9)	36 (52.9)
	Female	16 (42.1)	16 (53.3)	11 (61.1)	32 (47.1)
ECOG performance status, *n* (%)	0	37 (97.4)	25 (83.3)	14 (77.8)	62 (91.2)
	1	1 (2.6)	5 (16.7)	4 (22.2)	6 (8.8)
Lactate dehydrogenase, *n* (%)	>ULN	23 (60.5)	15 (50.0)	8 (44.4)	38 (55.9)
	≤ULN	15 (39.5)	15 (50.0)	10 (55.6)	30 (44.1)
Prior systemic therapies, *n* (%)	0	3 (7.9)	6 (20.0)	6 (33.3)	9 (13.2)
	1	19 (50.0)	18 (60.0)	9 (50.0)	37 (54.4)
	2	12 (31.6)	2 (6.7)	2 (11.1)	14 (20.6)
	>2	4 (10.5)	4 (13.3)	1 (5.6)	8 (11.8)
Prior immunotherapeutic agent, *n* (%)	All	22 (57.9)	14 (46.7)	4 (22.2)	36 (52.9)
	Ipilimumab	8 (21.1)	4 (13.3)	0	12 (17.6)
	Nivolumab	8 (21.1)	4 (13.3)	0	12 (17.6)
	Pembrolizumab	11 (28.9)	10 (33.3)	4 (22.2)	21 (30.9)
	Tebentafusp	0	0	0	0
Sites of metastases, *n* (%)	Liver only	13 (34.2)	14 (46.7)	9 (50.0)	27 (39.7)
	Liver + other	20 (52.6)	13 (43.3)	7 (38.9)	33 (48.5)
	Other	5 (13.2)	3 (10.0)	2 (11.1)	8 (11.8)
Maximum target lesion diameter (mm)	Median (range)	39 (11–126)	35.5 (11–201)	40 (11–201)	36 (11–201)

*BID* twice a day, *ECOG* Eastern Cooperative Oncology Group, *QD* once daily, *RDE* recommended dose for expansion, *ULN* upper limit of normal.

Overall, 59 patients (86.8%) had received prior systemic therapy; the majority having received either one (*n* = 37, 54.4%) or two prior regimens (*n* = 14, 20.6%). Eight patients received ≥3 prior regimens. Overall, few were reported to have responded to any prior therapy. The majority of patients (*n* = 31, 45.6%) had PD as the best overall response (BOR) to their last prior therapy and in these patients, the median time from initiation to discontinuation of last prior therapy was 63 days (range: 14–515). Approximately half of all patients (*n* = 36, 52.9%) had received a prior immunotherapeutic agent (CTLA-4 and/or PD-1 monoclonal antibodies). Of these 36 patients, 22 progressed, 10 had SD as their best response, 1 had a minor response, and the response was unknown in 3 patients. Other commonly received prior therapies included nitrosoureas (*n* = 20, 29.4%) and other alkylating agents (n = 13, 19.1%). No patients had received prior tebentafusp.

### Dose escalation and MTD declaration

In the dose escalation part of the study, 56 patients received LXS196 orally in one of two dosing schedules, either QD (*n* = 38) or BID (*n* = 18). Fifty-five patients were evaluable for MTD/RDE determination, with one patient excluded from the DLT analysis due lack of sufficient exposure to study treatment during cycle 1. Patients were initially treated on a QD schedule (100–1000 mg QD); however, due to toxicities reported at doses ≥500 mg QD, a BID schedule was then tested (200–400 mg BID). The MTDs were declared at 500 mg QD and 400 mg BID, based on the BLRM and EWOC principles. First cycle DLTs were observed in 7/38 (18.4%) and 2/17 (11.8%) patients (QD and BID, respectively). Following the BLRM recommendation guided by EWOC and review of all available safety and PK data, the RDE was declared at 300 mg BID (Supplementary Fig. 1).

### Safety and tolerability of LXS196

Of the 55 patients evaluable for MTD/RDE determination, DLTs were observed in nine (16.4%) patients (seven patients treated in the QD schedule and two patients in the BID schedule) (Table 2A). The most common DLT was hypotension (*n* = 6; 10.9%). In the QD schedule, hypotension leading to drug interruption and subsequent dose reduction occurred in five patients treated at doses ≥500 mg (two grade 3 events at 500 mg QD, one grade 4 event at 800 mg QD, one grade 4 and one grade 2 event at 1000 mg QD). These events most commonly occurred within 1 to 4 h after the first or second dose and were sometimes accompanied by a transient loss of consciousness with preceding dizziness and sweating. No corresponding tachycardia or ECG changes were observed clinically in the patients who experienced symptomatic hypotension and there were no signs of an allergic reaction such as skin rash, itching, wheezing, dyspnoea, or oedema. Most events resolved within minutes, some within 4 h, with an infusion of intravenous fluids and 2 patients experienced recurrence of hypotension with 1 patient requiring further LXS196 dose reduction.

**Table 2:** (A): Dose-limiting toxicities. (B): Adverse events, suspected to be study drug related by preferred term. (C): Grade ≥ 3 serious adverse events, regardless of study drug relationship by preferred term.

Table 2(A)	Dose-limiting toxicities											
DLT events, *n* (%)	LXS196 QD schedule							LXS196 BID schedule				All patients *n* = 55
	100 mg *n* = 3	200 mg *n* = 4	300 mg *n* = 15	500 mg *n* = 11	800 mg *n* = 1	1000 mg *n* = 4	All QD *n* = 38	200 mg *n* = 5	300 mg *n* = 6	400 mg *n* = 6	All BID *n* = 17	
Hypotension	0	0	0	2 (18.2) (Grade 3)	1 (100) (Grade 4)	2 (50.0) (Grade 2 and 4)	5 (13.2)	0	0	1 (16.7)	1 (5.9)	6 (10.9)
Nausea	0	1 (25.0) (Grade 3)	0	0	0	0	1 (2.6)	0	0	0	0	1 (1.8)
Vomiting	0	1 (25.0) (Grade 3)	0	0	0	0	1 (2.6)	0	0	0	0	1 (1.8)
Neutropenia	0	0	0	1 (9.1) (Grade 4)	0	0	1 (2.6)	0	0	0	0	1 (1.8)
Generalised oedema	0	0	0	0	0	0	0	0	0	1 (16.7) (Grade 3)	1 (5.9)	1 (1.8)
Total	0	2 (50.0)	0	3 (27.3)	1 (100)	2 (50.0)	8 (21.0)	0	0	2 (33.4)	2 (11.8)	9 (16.4)

Table 2(B)	Adverse events, suspected to be study drug related by preferred term^a^							
Preferred term, *n* (%)	All LXS196 QD patients *n* = 38		All LXS196 BID patients *n* = 30		LXS196 300 mg BID (RDE) patients *n* = 18		All LXS196 patients *N* = 68	
	All grades	Grade ≥ 3	All grades	Grade ≥ 3	All grades	Grade ≥ 3	All grades	Grade ≥ 3
Number of patients with ≥ 1 adverse event	35 (92.1)	11 (28.9)	28 (93.3)	6 (20.0)	17 (94.4)	2 (11.1)	63 (92.6)	17 (25.0)
Nausea	26 (68.4)	1 (2.6)	19 (63.3)	0	14 (77.8)	0	45 (66.2)	1 (1.5)
Diarrhoea	15 (39.5)	0	16 (53.3)	1 (3.3)	11 (61.1)	1 (5.6)	31 (45.6)	1 (1.5)
Vomiting	12 (31.6)	1 (2.6)	9 (30.0)	0	7 (38.9)	0	21 (30.9)	1 (1.5)
Alanine aminotransferase increased	7 (18.4)	2 (5.3)	8 (26.7)	2 (6.7)	5 (27.8)	1 (5.6)	15 (22.1)	4 (5.9)
Hypotension	8 (21.1)	5 (13.2)	7 (23.3)	1 (3.3)	3 (16.7)	0	15 (22.1)	6 (8.8)
Fatigue	10 (26.3)	0	4 (13.3)	0	3 (16.7)	0	14 (20.6)	0
Asthenia	6 (15.8)	0	7 (23.3)	0	4 (22.2)	0	13 (19.1)	0
Aspartate aminotransferase increased	5 (13.2)	2 (5.3)	6 (20.0)	2 (6.7)	3 (16.7)	1 (5.6)	11 (16.2)	4 (5.9)
Dry skin	2 (5.3)	0	6 (20.0)	0	4 (22.2)	0	8 (11.8)	0
Rash	3 (7.9)	0	5 (16.7)	0	4 (22.2)	0	8 (11.8)	0
Blood creatinine increased	2 (5.3)	0	5 (16.7)	0	2 (11.1)	0	7 (10.3)	0
Constipation	4 (10.5)	0	3 (10.0)	0	1 (5.6)	0	7 (10.3)	0

Table 2(C)	Grade ≥ 3 serious adverse events, regardless of study drug relationship by preferred term											
Preferred term, *n* (%)	LXS196 100 mg QD	LXS196 200 mg QD	LXS196 300 mg QD	LXS196 500 mg QD	LXS196 800 mg QD	LXS196 1000 mg QD	All LXS196 QD patients	LXS196 200 mg BID patients	LXS196 300 mg BID (RDE) patients	LXS196 400 mg BID patients	All LXS196 BID patients	All LXS196 patients
	*n* = 3	*n* = 4	*n* = 15	*n* = 11	*n* = 1	*n* = 4	*n* = 38	*n* = 6	*n* = 18	*n* = 6	*n* = 30	*N* *=* *68*
Number of patients with ≥ 1 adverse event	0	1 (25.0)	0	3 (27.3)	1 (100)	2 (50.0)	7 (18.4)	1 (16.7)	5 (27.8)	1 (16.7)	7 (23.3)	14 (20.6)
Hypotension	0	0	0	3 (27.3)	1 (100)	1 (25.0)	5 (13.2)	0	0	1 (16.7)	1 (3.3)	6 (8.8)
Pneumonia	0	1 (25.0)	0	0	0	1 (25.0)	2 (5.3)	0	1 (5.6)	0	1 (3.3)	3 (4.4)
Acute kidney injury	0	0	0	0	0	0	0	0	1 (5.6)	0	1 (3.3)	1 (1.5)
Cellulitis	0	0	0	0	0	0	0	1 (16.7)	0	0	1 (3.3)	1 (1.5)
Constipation	0	0*	0	0	0	0	0^b^	0	0	0	0	0^b^
Diarrhoea	0	0	0	0^b^	0	0	0^b^	0	0	0	0	0^b^
Eyelid bleeding	0	0	0	0	0	0	0	0	1 (5.6)	0	1 (3.3)	1 (1.5)
Hepatic failure	0	0	0	0	0	0	0	0	1 (5.6)	0	1 (3.3)	1 (1.5)
Hepatic cytolysis	0	0	0	0	0	0	0	0	1 (5.6)	0	1 (3.3)	1 (1.5)
Nausea	0	1 (25.0)	0	0	0	0	1 (2.6)	0	0	0	0	1 (1.5)
Neutrophil count decreased	0	0	0	0	0	0	0	0	1 (5.6)	0	1 (3.3)	1 (1.5)
Oedema peripheral	0	0	0	0	0	0	0	0	1 (5.6)	0	1 (3.3)	1 (1.5)
Rash pruritic	0	0	0	0	0	0	0	0	1 (5.6)	0	1 (3.3)	1 (1.5)
Thrombocytopenia	0	0	0	0	0	0	0	0	1 (5.6)	0	1 (3.3)	1 (1.5)
Vomiting	0	1 (25.0)	0	0	0	0	1 (2.6)	0	0	0	0	1 (1.5)

Table **2(A)**: *BID* twice a day, *DLT* dose-limiting toxicity, *QD*, once daily.

Table **2(B)**: A patient with multiple severity grades for an adverse event is only counted under the maximum grade. MedDRA version 24.1, CTCAE version 4.03.

*BID* twice a day, CTCAE common terminology criteria for adverse events, MedDRA medical dictionary for regulatory activities, *QD* once daily, *RDE* recommended dose for expansion.

^a^All grade adverse events occurring in ≥10% “All LXS196 patients”.

Table **2(C)**: All serious adverse events were CTCAE grade ≥3 with an exception noted below.

^b^Two patients experienced CTCAE grade 1/2 events of constipation/diarrhoea, which were reported as serious adverse events.

A patient with multiple occurrences of an AE under one treatment is counted only once in the adverse event category for that treatment.

A patient with multiple adverse events is counted only once in the total row.

*BID* twice a day, *CTCAE* common terminology criteria for adverse events, *QD* once daily, *RDE* recommended dose for expansion.

Following determination of the MTD in the QD dosing schedule, a BID schedule was introduced to explore whether alternative dosing may lead to better tolerability of LXS196. One DLT of grade 3 hypotension was reported in a patient treated at the highest BID dose tested (400 mg BID). The onset, characteristics, and resolution of this event were very similar to those reported in patients treated with the QD schedule.

Other DLTs included nausea and vomiting, neutropenia and generalised oedema (one patient each; 1.8%). GI-related DLTs resolved with temporary interruption of study drug and treatment with anti-emetics.

The majority of patients (*n* = 66; 97.1%) experienced an AE regardless of study drug relationship during the study; 29 (42.6%) of these patients experienced AEs of grade 3/4 (Supplementary Table 2). The most frequent AEs with LXS196 (all grades, all doses, both schedules) were nausea (*n* = 48; 70.6%), diarrhoea (*n* = 37; 54.4%), vomiting (*n* = 26; 38.2%), fatigue (*n* = 19; 27.9%), increased alanine aminotransferase (ALT, *n* = 18; 26.5%), constipation (*n* = 17; 25%), asthenia (*n* = 15; 22.1%) and hypotension (*n* = 15; 22.1%). All other AEs occurred in <20% of patients. The most frequent AEs of grade ≥3, regardless of study drug relationship, were hypotension (*n* = 6; 8.8%), increased aspartate aminotransferase (AST, *n* = 5; 7.4%) and increased ALT (*n* = 4; 5.9%). Overall, the type, incidence and severity of AEs regardless of study drug relationship were similar to those reported as suspected to be related to LXS196. The safety profile of the RDE (300 mg BID) is presented in Table 2B and Supplementary Table 3.

AEs suspected to be related to study treatment occurred in 63 (92.6%) patients; of these, 17 (25.0%) experienced grade 3/4 AEs. The most frequent AEs (occurring in ≥10% of patients) included nausea (*n* = 45; 66.2%), diarrhoea (*n* = 31; 45.6%), vomiting (*n* = 21; 30.9%), increased ALT (*n* = 15; 22.1%), hypotension (*n* = 15; 22.1%), fatigue (*n* = 14; 20.6%), asthenia (*n* = 13; 19.1%), increased AST (*n* = 11; 16.2%), dry skin (*n* = 8; 11.8%), rash (*n* = 8; 11.8%), increased blood creatinine (*n* = 7; 10.3) and constipation (*n* = 7; 10.3%). The most frequently reported grade 3/4 AEs were hypotension (*n* = 6; 8.8%), increased ALT (*n* = 4; 5.9%) and increased AST (*n* = 4; 5.9%).

Serious AEs (SAEs) were observed in 14 (20.6%) patients, and all were grade 3/4; the most frequent were hypotension (*n* = 6; 8.8%) and pneumonia (*n* = 3; 4.4%) (Table 2C). BID appeared to be better tolerated than QD dosing, with fewer treatment-related grade 3/4 AEs and treatment-related SAEs. Grade 3/4 hypotension was more common in the QD versus the BID groups (13.2% versus 3.3%).

The only AE leading to discontinuation of LXS196 was an SAE of grade 3/4 hepatic failure in a patient treated in the BID schedule with known liver metastases and CTCAE grade 2 transaminases at study entry. The event was suspected to be related to underlying disease progression but was not confirmed by radiological assessment.

### Pharmacokinetics and pharmacodynamics

**Fig. 1:**
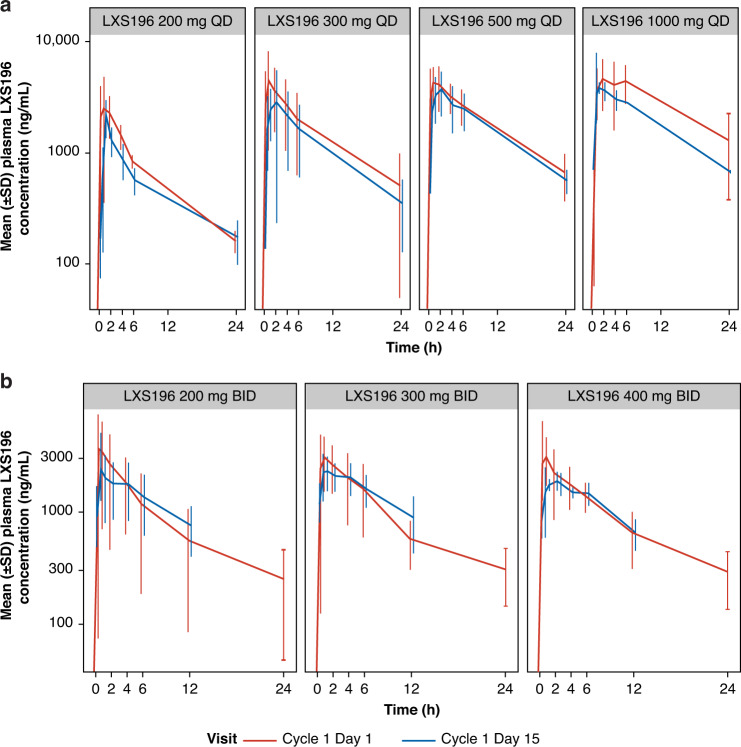
Arithmetic mean (SD) concentration-time profiles following administration of LXS196 on cycle 1 day 1 and cycle 1 day 15 following multiple oral doses **a** LXS196 200–1000 mg QD and **b** LXS196 200–400  mg BID. BID twice a day, C1D1 cycle 1 day 1, C1D15 cycle 1 day 15, QD once daily, SD standard deviation.

Plasma concentration profiles on C1D1 and C1D15 are shown in Fig. 1 and PK parameters are presented in Supplementary Table 4. Following oral dosing of LXS196, the median time to reach *T*_max_ ranged from 0.483–2.00 h on both D1 and D15. The mean terminal half-life (*T*_1/2_) was consistent across tested dose groups ranging from 8.49–13.8 h. Minimal or reduced accumulation was observed with repeated administration (mean *R*_acc_ ranged from 0.718 to 1.27-fold). PK variability was moderate, as illustrated by the between-patient variability (CV%) for *C*_max_ and for AUC_0–*t*_. Increase in exposure (AUC_0–*t*_ and *C*_max_) on C1D1 and D15 was under-proportional to the increase in dose with both QD and BID schedules.

**Fig. 2:**
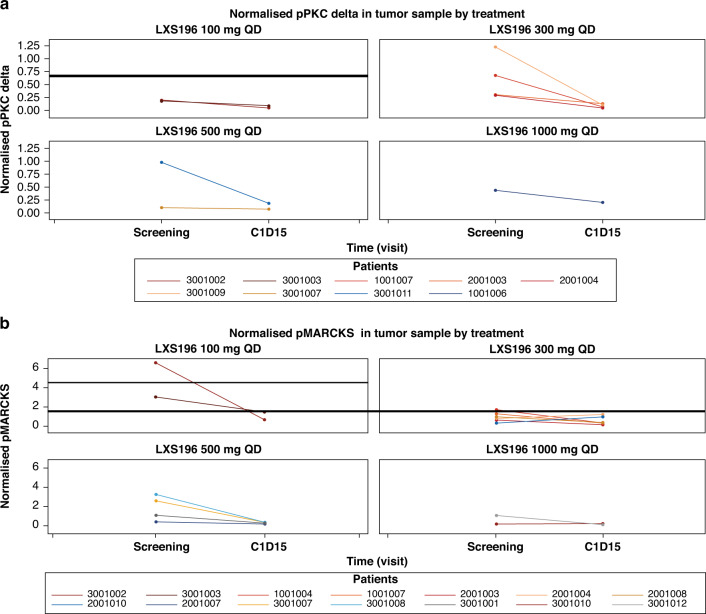
pMARKS and pPKCdelta reduction in tumor samples following administration of LXS196. **a** Normalised pPKC delta and **b** Normalised pMARCKS in tumour sample by treatment. Note: Normalisation calculations for pPKC and pMARCK are detailed in the supplementary appendix. C1D15, cycle 1 day 15; pMARCKS phosphorylated myristoylated alanine-rich C-kinase substrate, pPKC phosphorylated protein kinase C delta; QD, once daily.

LXS196 reduced pMARCKS and phosphorylated PKC delta (pPKC delta), suggesting target engagement in on-treatment tumour biopsies. Reduction in pPKC delta in PBMCs was observed within 6 h post-dose at all dose levels (Fig. 2a, b). The mean (range) reduction in percentage change from baseline to C1D15 (normalised ratio) for pPKC delta and pMARCKS was −68.1 (−91.4, −28.6) and −39.7 (−89.7, 199), respectively.

### Efficacy

**Fig. 3:**
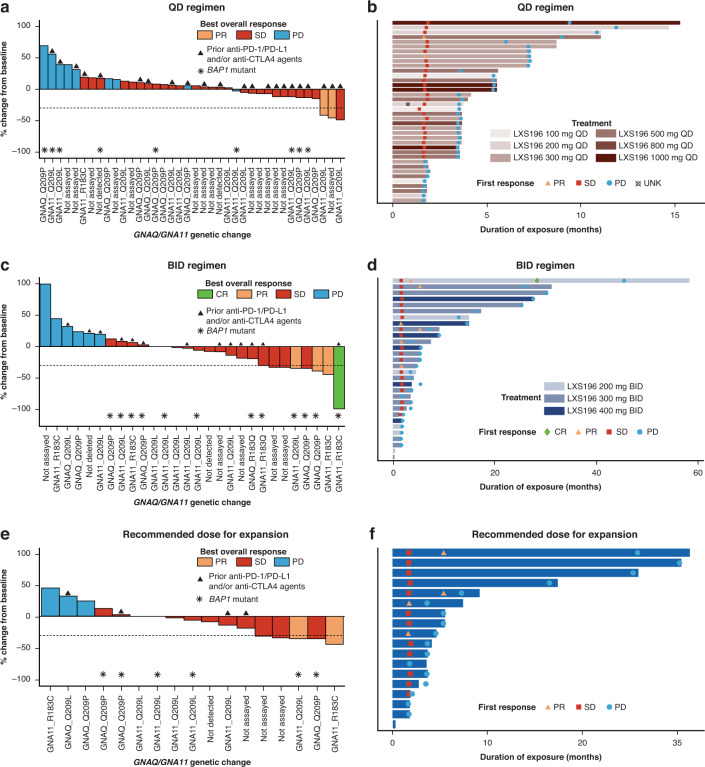
Waterfall plot for best percentage change from baseline in sum of longest diameters based on local radiology review and swimmer plot for duration of exposure. Figures 3a, c and e present waterfall plots for QD, BID, and RDE (300 mg BID) regimens, respectively. Patients with a best percentage change of ≥30% decrease from baseline in sum of longest diameters, which was not confirmed at the next scheduled evaluation were assessed as having a BOR of SD by the investigator per RECIST v1.1. Figure 3b, d and f present swimmer plots for QD, BID, and RDE (300 mg BID) regimens, respectively. The bars show the duration of exposure to study treatment. The first overall response of SD, unconfirmed PR, CR, or PD for each patient, as reported by the investigator per RECIST v1.1, is represented by a square, triangle, diamond, and circle, respectively. BID twice a day, BOR best overall response, CR complete response, CTLA4 cytotoxic T-lymphocyte-associated protein 4, PD progressive disease, PD-1 programmed cell death 1, PD-L1 programmed cell death ligand 1, PR partial response, QD once daily, RDE recommended dose for expansion, RECIST response evaluation criteria in solid tumours, SD stable disease, UNK unknown.

As of the final data cut-off date (7 January 2022), a total of 68 patients had been treated with LXS196 and the median duration of exposure was 3.71 months (range: 1.81–15.28) and 4.6 months (range: 0.33–58.32) for patients in the QD and BID regimens, respectively. Overall, 66 patients had completed at least 1 post-baseline assessment as per RECIST v1.1 and were considered evaluable for response; 1 had a complete response (CR; BID group), 5 had partial response (PR) (2 in the QD group and 3 in the BID group) and 45 had SD as their BOR. The median duration of response for all patients with a RECIST response (*n* = 6) was 10 months (2.99–41.95) and median duration of SD (*n* = 45) was 5.32 months (2.07–30.23). Of the 30 patients treated in the BID schedule, 4 (13.3%) had a BOR of CR or PR, and 18 (60%) had SD. The ORR in the BID schedule was 13.3% (95% CI: 3.8–30.7) and the disease control rate (DCR) was 73.3% (95% CI: 54.1–87.7) (Table 3). Of the 18 evaluable patients treated at the RDE of 300 mg BID, 2 (11.1%) had a BOR of PR and 12 (66.7%) had SD (including 3 patients with >30% tumour reduction/unconfirmed PR). The ORR at the RDE was 11.1% (95% CI: 1.4–34.7) and the DCR was 77.8% (95% CI: 52.4–93.6) (Fig. 3).

**Table 3:** Best overall response and progression-free survival.

	All LXS196 QD patients *n* = 38	All LXS196 BID patients *n* = 30	LXS196 300 mg BID (RDE) patients *n* = 18	All LXS196 patients *N* = 68
Best overall response (BOR), *n* (%)				
Complete response (CR)	0	1 (3.3)	0	1 (1.5)
Partial response (PR)	2 (5.3)	3 (10.0)	2 (11.1)	5 (7.4)
Stable disease (SD)	27 (71.1)	18 (60.0)	12 (66.7)	45 (66.2)
Progressive disease (PD)	9 (23.7)	6 (20.0)	3 (16.7)	15 (22.1)
Unknown^a^	0	2 (6.7)	1 (5.6)	2 (2.9)
Overall response rate (ORR: CR + PR), *n* (%) [95% CI]	2 (5.3) [0.6–17.7]	4 (13.3) [3.8–30.7]	2 (11.1) [1.4–34.7]	6 (8.8) [3.3–18.2]
Disease control rate (DCR: CR + PR + SD), *n* (%) [95% CI]	29 (76.3) [59.8–88.6]	22 (73.3) [54.1–87.7]	14 (77.8) [52.4–93.6]	51 (75.0) [63–84.7]
Progression-free survival (PFS), *n* (%)				
Number of PFS events	38 (100.0)	30 (100)	18 (100)	68 (100)
Progression	38 (100.0)	28 (93.3)	17 (94.4)	66 (97.1)
Deaths	0 (0.0)	2 (6.7)	1 (5.6)	2 (2.9)
PFS percentiles (95% CI)				
25th	3.4 (1.7–3.5)	1.9 (1.6–3.6)	2.1 (0.8–3.6)	2.0 (1.7–3.5)
50th	3.5 (3.5–5.4)	4.1 (3.5–7.2)	3.7 (2.1–5.4)	3.6 (3.5–5.4)
75th	6.0 (3.6–7.3)	12.9 (5.4–25.8)	7.2 (3.7–25.8)	7.2 (5.4–9.4)
Kaplan–Meier estimates (%) PFS rate (95% CI) at				
4 months	36.8 (22.0–51.8)	50.0 (31.3–66.1)	44.4 (21.6–65.1)	42.6 (30.8–54.0)
6 months	26.3 (13.7–40.8)	33.3 (17.5–50.0)	27.8 (10.1–48.9)	29.4 (19.1–29.2)
12 months	0.0 (NE–NE)	26.7 (12.6–43.0)	22.2 (6.9–42.9)	11.8 (5.5–20.6)

BOR is based on investigator’s assessment using RECIST v1.1.

Estimate [95% CI] for ORR and DCR were obtained using Clopper and Pearson’s method.

PFS probability estimates are obtained from the Kaplan–Meier survival estimates; Greenwood formula is used for CIs.

Percentiles with 95% CIs are calculated from PROC LIFETEST output.

^a^Two patients did not have post-baseline tumour assessments due to clinical disease progression before the first scheduled assessment. Hence, the BOR is unknown per RECIST 1.1, however, they are included in the denominator for ORR and DCR calculations.

*BID* twice a day, *CI* confidence interval, *NE* not estimable, *QD* once daily, *RECIST* response evaluation criteria in solid tumours, *RDE* recommended dose for expansion.

No patients were censored and 100% of PFS events were reported as of the data cut-off date. Of the 68 events, 66 had disease progression and 2 patients had died. The estimated median PFS was 3.6 months (95% CI: 3.5–5.4) and the estimated PFS rate at 6 months by the KM method was 29.4% (95% CI: 19.1–40.4). In the BID schedule, the estimated median PFS was 4.1 months (95% CI: 3.5–7.2) and the estimated PFS rate at 6 months by the KM method was 33.3% (95% CI: 17.5–50). At the RDE of 300 mg BID, the estimated median PFS was 3.7 months (95% CI: 2.1–5.4) and the estimated PFS rate at 6 months by the KM method was 27.8% (95% CI: 10.1–48.9) (Table 3).

### DNA sequencing analysis

Patients (48/68) were assessed for somatic DNA mutations using a targeted sequencing panel (Yap 2018); 74% of tumour biopsies were from liver metastases. Mutations in the *GNAQ* and *GNA11* genes are tabulated (Supplementary Table 5A); of the 48 patients assessed, 28 were mutant in *GNA11* (of which 22 had allele Q209L and 6 had R183C), 16 were mutant in *GNAQ* (of which 13 had allele Q209P, two Q209L, and one R183Q), and 4 were not called mutant in either gene as the allele fraction was below the limit of detection. The genes CYSLTR2 and PLCB4 were not included in the sequencing panel. The 92% *GNAQ/GNA11* mutation rate is consistent with previous findings, as is the observation that *GNAQ* and *GNA11* mutations were mutually exclusive [13, 36]. No association of *GNAQ/GNA11* mutation status with patient response was observed (Fig. 3). *BAP1* mutations resulting in a protein coding change were detected in 44% (21/48) of patients (Supplementary Table 5B).

### Gene expression analysis

RNAseq was used to explore gene expression in 68 patients at screening and on treatment at C1D15 ± 3 days. RasGRP3 was decreased in some patients receiving LXS196 both QD and BID (Supplementary Fig. 2). Levels of RasGRP3 at screening and after treatment also correlate with the best percent change in tumour volume from baseline (Supplementary Fig. 2). MAPK pathway activity as measured by DUSP6 expression showed a trend towards suppression in the PR group (Supplementary Fig. 2).

## Discussion

In this study, we explored the possibility of targeting *GNAQ/GNA11* and other G-protein pathway-associated mutations by inhibiting the downstream effector PKC using LXS196.

The patients enroled in this first-in-human phase I study displayed characteristics representative of the MUM patient population. Liver metastases were present in 88% of the patients, and baseline LDH was >ULN in 56%. The majority of patients (86.8%) had received prior systemic therapy, including immunotherapy in 53%, and had not achieved a response to prior therapy.

Overall, LXS196 was generally well tolerated as a single agent, with the majority of patients not requiring interruption of LXS196 or a dose reduction from their assigned dose level. Non-clinical data suggested patients may experience a decrease in systolic blood pressure, and indeed hypotension was observed in a number of patients on study at doses ≥500 mg QD. All patients experiencing symptomatic hypotension achieved complete recovery within minutes to a few hours, some following infusion of intravenous fluids. Active management of symptomatic hypotension was recommended per protocol and regular vital signs (including blood pressure and heart rate) and ECG monitoring were required throughout the study at regular study visits. Investigators were encouraged to temporarily withhold concomitant medications that may cause hypotension prior to the first dose of LXS196 and implement more frequent blood pressure monitoring throughout the study as deemed necessary per clinical need. The exact mechanism by which PKC inhibition may lead to hypotension warrants further investigation, however, as PKC is known to play a role in vascular smooth muscle (VSM) function, one possible hypothesis is that inhibition of PKCα could lead to a decrease in mean arterial pressure by a reduction in vascular smooth muscle contractility [37–39].

The most frequent AEs suspected to be related to LXS196 in patients across both dosing schedules included nausea, diarrhoea, vomiting, hypotension, increased ALT and fatigue. Most GI and constitutional AEs were mild to moderate (grade 1/2) and manageable. BID dosing was better tolerated than QD dosing, with fewer grade 3/4 AEs reported and fewer drug-related SAEs. In this study, MTDs were determined to be 500 mg QD and 400 mg BID, and the RDE was declared to be 300 mg BID.

During the dose escalation part of the study, following oral doses of 100–1000 mg QD and 200–400 mg BID, total plasma concentration profiles of LXS196 showed rapid absorption in fasting conditions with a *T*_max_ of ~1 h after dose (median *T*_max_ ranging from 0.483–2.00 h) and consistent terminal *T*_1/2_ across different doses (~11 h). There was minimal or reduced accumulation of LXS196 with repeated dose. PK variability was moderate. Dose proportionality analysis showed an under-proportional increase in exposure with dose for both QD and BID schedules.

LXS196 reduces pMARCKS and pPKCd, suggesting target engagement in on-treatment tumour biopsies. A decrease in pMARCKS/MARCKS was observed by C1D15 in patient tumour samples; however, no clear associations were detected between the extent of pMARCKS suppression and LXS196 treatment group. Consistent with this, a decrease in pPKCd was observed by C1D15, but no correlation was seen between the extent of suppression and the LXS196 treatment group. The decrease in pPKCd was also observed in PBMCs, which are not expected to carry mutations in the *GNAQ/GNA11* pathway. There are several possible explanations for the lack of correlation between reductions in pMARKCS/pPKCd and clinical activity. One possibility is that the PKC signalling pathway is highly sensitive to LXS196 exposure, and a C1D15 timepoint might be too late to observe transient changes. Interrogation at an earlier timepoint to determine pathway modulation might result in better association with the dose of LSX196 and/or clinical outcome.

Exploratory analysis of RasGRP3 reveals a potential biomarker for predicting efficacy and evidence of a transcriptional regulatory feedback on RasGRP3 by PKC inhibition and warrants further investigation.

Compared to the first-generation PKC inhibitor AEB071 [12], LXS196 was well tolerated and showed promising clinical activity. As with AEB071, commonly reported toxicities included GI AEs (nausea, vomiting, diarrhoea); however, dose-related symptomatic hypotension was commonly reported in this first-in-human study of LXS196, an AE not observed with the first-generation PKC inhibitor. Events of hypotension were manageable, and most patients did not require treatment interruption or dose reduction. Regarding efficacy, across all doses tested the ORR was 8.8% with LXS196 versus 3% with AEB071, and 67% of patients had SD as their best response with LXS196 versus 50% with AEB071 (median duration of SD: 5.32 versus 3.75 months, respectively). The estimated median PFS was similar at 3.6 months (95% CI: 3.5–5.4) with LXS196 versus 3.5 months (95% CI: 2.5–3.6) with AEB071.

## Conclusion

These results confirm the tolerable safety profile of LXS196. The encouraging clinical activity demonstrated provides evidence that targeting the PKC pathway in MUM should be explored and supports the continued evaluation of LXS196 in combination with other targeted therapies. LXS196, now known as darovasertib (IDE196), is currently being explored by Ideaya biosciences in doublet combinations with the MEK inhibitor binimetinib and the cMET inhibitor crizotinib (NCT03947385).

## Supplementary information

Supplementary Appendix

Reporting checklist

## Data Availability

Novartis will not provide access to patient-level data, if there is a reasonable likelihood that individual patients could be re-identified. Phase 1 studies, by their nature, present a high risk of patient re-identification; therefore, patient’s individual results for phase 1 studies cannot be shared. In addition, clinical data, in some cases, have been collected subject to contractual or consent provisions that prohibit transfer to third parties. Such restrictions may preclude granting access under these provisions. Where co-development agreements or other legal restrictions prevent companies from sharing particular data, companies will work with qualified requestors to provide summary information where possible.
