# Supplementary material for: A phase I trial of LXS196, a protein kinase C (PKC) inhibitor, for metastatic uveal melanoma
Source: Br J Cancer. 2023 Jan 9;128(6):1040–51. doi: 10.1038/s41416-022-02133-6 (PMC10006169; doi:10.1038/s41416-022-02133-6)
Supplement: Supplementary file 1 — Supplementary Appendix [file 41416_2022_2133_MOESM1_ESM.docx]

**Supplementary Appendix**

**Methods:**

**Sequencing data generation and analysis**

DNA and RNA were extracted from tumour tissue and analysed for nucleic acid quantity (Qubit®) and quality (Agilent Genomic DNA and RNA ScreenTape assays^®^, Agilent 2200 TapeStation System) using DNA and RNA integrity numbers (DINs and RINs, respectively) [1, 2]. DNA libraries were generated using the TruSeq Nano Library Preparation kit (Illumina). Hybridisation capture to a customised Agilent SureSelectXT panel was used to enrich coding regions from 567 cancer-related genes. The NGDx PanCancer version 3 panel interrogates the entire coding regions of 567 cancer-related genes plus select introns from 57 genes often rearranged or altered in solid tumour cancers [3]. Sequence reads, aligned with BWA-MEM [4] to the hg19 human reference genome, were marked for PCR duplicates with Picard, and had base quality scores recalibrated with the Genome Analysis ToolKit [5, 6]. Single nucleotide variants (SNVs) were called using MuTect [7], insertions and deletions (indels) were called with Pindel [8], translocations were called with Socrates [9] and tumour purity and copy number alterations were estimated using PureCN [10].

RNA libraries were prepared for RNAseq as previously described [11]. Briefly, the total RNA was depleted of ribosomal RNA (rRNA), fragmented, converted to cDNA, and then the next generation sequencing (NGS) library was constructed using the TruSeq RNA v2 Library Preparation Kit (Illumina) with end repair, A-tailing, indexed adaptor ligation and PCR amplification steps. Sequence reads were aligned with STAR [12] to the hg19 human reference genome, and read counts of coding regions were summarised by HTSeq [13] using a reference transcriptome of human Refseq transcripts.

**References:**

1. Technologies A: Agilent Genomic DNA ScreenTape System Quick Guide. (p/n G2964-90040). . In:2014.
2. Braly L, Brohawn P, Higgins P, Albright CA, Boland JF. Advancing the quality control methodology to asses isolated total RNA and generated fragmented cRNA. Agilent Appl Note Publ Number. 2003:5988-9861EN.
3. Yap Y-S, Singh AP, Lim JHC, Ahn J-H, Jung K-H, Kim J, et al. Elucidating therapeutic molecular targets in premenopausal Asian women with recurrent breast cancers. npj Breast Cancer. 2018;4:19.
4. Li H. Aligning sequence reads, clone sequences and assembly contigs with BWA-MEM. arXiv preprint arXiv:13033997. 2013.
5. McKenna A, Hanna M, Banks E, Sivachenko A, Cibulskis K, Kernytsky A, et al. The Genome Analysis Toolkit: a MapReduce framework for analyzing next-generation DNA sequencing data. Genome Res. 2010;20:1297-1303.
6. DePristo MA, Banks E, Poplin R, Garimella KV, Maguire JR, Hartl C, et al. A framework for variation discovery and genotyping using next-generation DNA sequencing data. Nat Genet. 2011;43:491-498.
7. Cibulskis K, Lawrence MS, Carter SL, Sivachenko A, Jaffe D, Sougnez C, et al. Sensitive detection of somatic point mutations in impure and heterogeneous cancer samples. Nature Biotechnology. 2013;31:213-219.
8. Ye K, Schulz MH, Long Q, Apweiler R, Ning Z. Pindel: a pattern growth approach to detect break points of large deletions and medium sized insertions from paired-end short reads. Bioinformatics. 2009;25:2865-2871.
9. Schröder J, Hsu A, Boyle SE, Macintyre G, Cmero M, Tothill RW, et al. Socrates: identification of genomic rearrangements in tumour genomes by re-aligning soft clipped reads. Bioinformatics. 2014;30:1064-1072.
10. Riester M, Singh AP, Brannon AR, Yu K, Campbell CD, Chiang DY, et al. PureCN: copy number calling and SNV classification using targeted short read sequencing. Source Code Biol Med. 2016;11:13.
11. Adiconis X, Borges-Rivera D, Satija R, DeLuca DS, Busby MA, Berlin AM, et al. Comparative analysis of RNA sequencing methods for degraded or low-input samples. Nat Methods. 2013;10:623-629.
12. Dobin A, Davis CA, Schlesinger F, Drenkow J, Zaleski C, Jha S, et al. STAR: ultrafast universal RNA-seq aligner. Bioinformatics. 2013;29:15-21.
13. Anders S, Pyl PT, Huber W. HTSeq--a Python framework to work with high-throughput sequencing data. Bioinformatics. 2015;31:166-169.

**Normalisation of PKC delta data for analysis**

Total and phosphorylated PKC delta were measured in all feasible tumour biopsies. For the analysis, the peak area under the curve corresponding to the signal for total and phosphorylated PKC delta were measured and adjusted for the amount of total protein in the tumour lysate used. The normalised PKC delta is calculated as follows:

Normalised PKC delta = Adjusted total PKC delta / Adjusted phosphorylated PKC delta

**Normalisation of MARCKS data for analysis**

Total and phosphorylated MARCKS were measured in all feasible tumour biopsies. For the analysis, concentration of total and phosphorylated MARCKS were measured and adjusted for the amount of total protein in the tumour lysate used. The normalised MARCKS is calculated as follows:

Normalised MARCKS = Adjusted total MARCKS / Adjusted phosphorylated MARCKS

**Supplementary Tables and Figures**

**Supplementary Table 1: Patient disposition**

| **Disposition reason, n (%)** | **All LXS196 QD patients n=38** | **All LXS196 BID patients n=30** | **LXS196 300 mg BID (RDE) patients**  **n=18** | **All LXS196 patients  N=68** |
| --- | --- | --- | --- | --- |
| **Patients treated** | | | | |
| **Treatment discontinued** | 38 (100) | 30 (100) | 18 (100) | 68 (100) |
| **Primary reason for end of treatment** | | | | |
| **Death** | 0 | 2 (6.7) | 1 (5.6) | 2 (2.9)* |
| **Progressive disease** | 38 (100) | 28 (93.3) | 17 (94.4) | 66 (97.1) |
| **Dose interruptions** | | | | |
| **With at least one dose interruption** | 11 (28.9) | 6 (20) | 3 (16.7) | 17 (25) |
| **With more than one dose interruption** | 5 (13.2) | 3 (10.0) | 1 (5.6) | 8 (11.8) |
| **With only one dose interruption** | 6 (15.8) | 3 (10) | 2 (11.1) | 9 (13.2) |
| **Number of patients with at least one dose interruption by reason** | | | | |
| **Adverse event** | 11 (28.9) | 5 (16.7) | 3 (16.7) | 16 (23.5) |
| **Physician decision** | 1 (2.6) | 0 | 0 | 1 (1.5) |
| **Patient/guardian decision** | 0 | 1 (3.3) | 0 | 1 (1.5) |

*Death due to underlying disease progression.

BID, twice a day; QD, once daily; RDE, recommended dose for expansion.

**Supplementary Table 2: Summary of adverse events regardless of study drug relationship by preferred term**

| **Preferred term, n (%)** | **LXS196 100 mg QD patients** | | **LXS196 200 mg QD patients** | | **LXS196 300 mg QD patients** | | **LXS196 500 mg QD patients** | | **LXS196 800 mg QD patients** | | **LXS196 1000 mg QD patients** | | **All LXS196 QD**  **patients** | |
| --- | --- | --- | --- | --- | --- | --- | --- | --- | --- | --- | --- | --- | --- | --- |
|  | **n=3** | | **n=4** | | **n=15** | | **n=11** | | **n=1** | | **n=4** | | **n=38** | |
|  | **All grades** | **Grade ≥3** | **All grades** | **Grade ≥3** | **All grades** | **Grade ≥3** | **All grades** | **Grade ≥3** | **All grades** | **Grade ≥3** | **All grades** | **Grade ≥3** | **All grades** | **Grade ≥3** |
| **Number of patients with ≥1 adverse event** | **3 (100)** | **0** | **4 (100)** | **3 (75.0)** | **14 (93.3)** | **4 (26.7)** | **10 (90.9)** | **4 (36.4)** | **1 (100)** | **1 (100)** | **4 (100)** | **2 (50.0)** | **36 (94.7)** | **14 (36.8)** |
| Nausea | 2 (66.7) | 0 | 2 (50.0) | 1 (25.0) | 10 (66.7) | 0 | 8 (72.7) | 0 | 1 (100) | 0 | 4 (100) | 0 | 27 (71.1) | 1 (2.6) |
| Diarrhoea | 2 (66.7) | 0 | 2 (50.0) | 0 | 6 (40.0) | 0 | 5 (45.5) | 0 | 1 (100) | 0 | 4 (100) | 0 | 20 (52.6) | 0 |
| Vomiting | 2 (66.7) | 0 | 2 (50.0) | 1 (25.0) | 6 (40.0) | 0 | 5 (45.5) | 0 | 0 | 0 | 0 | 0 | 15 (39.5) | 1 (2.6) |
| Fatigue | 0 | 0 | 0 | 0 | 8 (53.3) | 0 | 1 (9.1) | 0 | 0 | 0 | 3 (75.0) | 0 | 12 (31.6) | 0 |
| Alanine aminotransferase increased | 0 | 0 | 1 (25.0) | 0 | 4 (26.7) | 1 (6.7) | 3 (27.3) | 1 (9.1) | 0 | 0 | 1 (25.0) | 0 | 9 (23.7) | 2 (5.3) |
| Constipation | 1 (33.3) | 0 | 3 (75.0) | 0 | 4 (26.7) | 0 | 2 (18.2) | 0 | 0 | 0 | 0 | 0 | 10 (26.3) | 0 |
| Asthenia | 0 | 0 | 1 (25.0) | 0 | 3 (20.0) | 0 | 4 (36.4) | 0 | 0 | 0 | 0 | 0 | 8 (21.1) | 0 |
| Hypotension | 0 | 0 | 0 | 0 | 1 (6.7) | 0 | 4 (36.4) | 3 (27.3) | 1 (100) | 1 (100) | 2 (50.0) | 1 (25.0) | 8 (21.1) | 5 (13.2) |
| Aspartate aminotransferase increased | 0 | 0 | 0 | 0 | 1 (6.7) | 1 (6.7) | 4 (36.4) | 1 (9.1) | 0 | 0 | 0 | 0 | 5 (13.2) | 2 (5.3) |
| Oedema peripheral | 1 (33.3) | 0 | 1 (25.0) | 0 | 2 (13.3) | 0 | 1 (9.1) | 0 | 0 | 0 | 0 | 0 | 5 (13.2) | 0 |
| Decreased appetite | 0 | 0 | 1 (25.0) | 0 | 0 | 0 | 4 (36.4) | 0 | 0 | 0 | 0 | 0 | 5 (13.2) | 0 |
| Rash | 0 | 0 | 0 | 0 | 4 (26.7) | 0 | 0 | 0 | 0 | 0 | 0 | 0 | 4 (10.5) | 0 |
| Dry skin | 0 | 0 | 0 | 0 | 1 (6.7) | 0 | 1 (9.1) | 0 | 0 | 0 | 1 (25.0) | 0 | 3 (7.9) | 0 |
| Blood creatinine increased | 0 | 0 | 1 (25.0) | 0 | 2 (13.3) | 0 | 0 | 0 | 0 | 0 | 0 | 0 | 3 (7.9) | 0 |
| Abdominal pain upper | 0 | 0 | 0 | 0 | 2 (13.3) | 0 | 2 (18.2) | 0 | 0 | 0 | 0 | 0 | 4 (10.5) | 0 |
| Dermatitis acneiform | 0 | 0 | 0 | 0 | 1 (6.7) | 0 | 1 (9.1) | 0 | 0 | 0 | 0 | 0 | 2 (5.3) | 0 |
| Nasopharyngitis | 0 | 0 | 0 | 0 | 3 (20.0) | 0 | 0 | 0 | 0 | 0 | 1 (25.0) | 0 | 4 (10.5) | 0 |
| Neutropenia | 0 | 0 | 0 | 0 | 3 (20.0) | 1 (6.7) | 2 (18.2) | 1 (9.1) | 0 | 0 | 0 | 0 | 5 (13.2) | 2 (5.3) |

| **Preferred term, n (%)** | **LXS196 200 mg BID patients** | | **LXS196 300 mg BID (RDE) patients** | | **LXS196 400 mg BID patients** | | **All LXS196 BID patients** | | **All LXS196  patients** | |
| --- | --- | --- | --- | --- | --- | --- | --- | --- | --- | --- |
|  | **n=6** | | **n=18** | | **n=6** | | **n=30** | | **N=68** | |
|  | **All grades** | **Grade ≥3** | **All grades** | **Grade ≥3** | **All grades** | **Grade ≥3** | **All grades** | **Grade ≥3** | **All grades** | **Grade ≥3** |
| **Number of patients with ≥1 adverse event** | **6 (100)** | **3 (50.0)** | **18 (100)** | **8 (44.4)** | **6 (100)** | **4 (66.7)** | **30 (100)** | **15 (50.0)** | **66 (97.1)** | **29 (42.6)** |
| Nausea | 3 (50.0) | 0 | 15 (83.3) | 0 | 3 (50.0) | 0 | 21 (70.0) | 0 | 48 (70.6) | 1 (1.5) |
| Diarrhoea | 3 (50.0) | 0 | 11 (61.1) | 1 (5.6) | 3 (50.0) | 0 | 17 (56.7) | 1 (3.3) | 37 (54.4) | 1 (1.5) |
| Vomiting | 0 | 0 | 8 (44.4) | 0 | 3 (50.0) | 0 | 11 (36.7) | 0 | 26 (38.2) | 1 (1.5) |
| Fatigue | 0 | 0 | 6 (33.3) | 0 | 1 (16.7) | 0 | 7 (23.3) | 0 | 19 (27.9) | 0 |
| Alanine aminotransferase increased | 1 (16.7) | 0 | 5 (27.8) | 1 (5.6) | 3 (50.0) | 1 (16.7) | 9 (30.0) | 2 (6.7) | 18 (26.5) | 4 (5.9) |
| Constipation | 1 (16.7) | 0 | 4 (22.2) | 0 | 2 (33.3) | 0 | 7 (23.3) | 0 | 17 (25.0) | 0 |
| Asthenia | 1 (16.7) | 0 | 4 (22.2) | 0 | 2 (33.3) | 0 | 7 (23.3) | 0 | 15 (22.1) | 0 |
| Hypotension | 0 | 0 | 3 (16.7) | 0 | 4 (66.7) | 1 (16.7) | 7 (23.3) | 1 (3.3) | 15 (22.1) | 6 (8.8) |
| Aspartate aminotransferase increased | 1 (16.7) | 0 | 4 (22.2) | 2 (11.1) | 3 (50.0) | 1 (16.7) | 8 (26.7) | 3 (10.0) | 13 (19.1) | 5 (7.4) |
| Oedema peripheral | 2 (33.3) | 0 | 5 (27.8) | 1 (5.6) | 1 (16.7) | 0 | 8 (26.7) | 1 (3.3) | 13 (19.1) | 1 (1.5) |
| Decreased appetite | 1 (16.7) | 0 | 2 (11.1) | 0 | 2 (33.3) | 0 | 5 (16.7) | 0 | 10 (14.7) | 0 |
| Rash | 0 | 0 | 5 (27.8) | 0 | 1 (16.7) | 0 | 6 (20.0) | 0 | 10 (14.7) | 0 |
| Dry skin | 1 (16.7) | 0 | 4 (22.2) | 0 | 1 (16.7) | 0 | 6 (20.0) | 0 | 9 (13.2) | 0 |
| Blood creatinine increased | 3 (50.0) | 0 | 2 (11.1) | 0 | 0 | 0 | 5 (16.7) | 0 | 8 (11.8) | 0 |
| Abdominal pain upper | 0 | 0 | 3 (16.7) | 0 | 0 | 0 | 3 (10.0) | 0 | 7 (10.3) | 0 |
| Dermatitis acneiform | 1 (16.7) | 0 | 3 (16.7) | 0 | 1 (16.7) | 0 | 5 (16.7) | 0 | 7 (10.3) | 0 |
| Nasopharyngitis | 1 (16.7) | 0 | 2 (11.1) | 0 | 0 | 0 | 3 (10.0) | 0 | 7 (10.3) | 0 |
| Neutropenia | 1 (16.7) | 0 | 0 | 0 | 1 (16.7) | 0 | 2 (6.7) | 0 | 7 (10.3) | 2 (2.9) |

*All grade adverse events occurring in ≥10% “All LXS196 patients”.

A patient with multiple severity grades for an adverse event is only counted under the maximum grade. MedDRA version 24.1, CTCAE version 4.03.

AE, adverse event; BID, twice a day; CTCAE, Common Terminology Criteria for Adverse Events; MedDRA, Medical Dictionary for Regulatory Activities; QD, once daily; RDE, recommended dose for expansion.

**Supplementary Table 3: Summary of adverse events suspected to be study drug related by preferred term***

| **Preferred term, n (%)** | **LXS196 100 mg QD** | | **LXS196 200 mg QD** | | **LXS196 300 mg QD** | | **LXS196 500 mg QD** | | **LXS196 800 mg QD** | | **LXS196 1000 mg QD** | | | **All LXS196 QD patients** | |
| --- | --- | --- | --- | --- | --- | --- | --- | --- | --- | --- | --- | --- | --- | --- | --- |
|  | **n=3** | | **n=4** | | **n=15** | | **n=11** | | **n=1** | | **n=4** | | | **n=38** | |
|  | **All grades** | **Grade ≥3** | **All grades** | **Grade ≥3** | **All grades** | **Grade ≥3** | **All grades** | **Grade ≥3** | **All grades** | **Grade ≥3** | **All grades** | **Grade ≥3** | **All grades** | | **Grade ≥3** |
| **Number of patients with ≥1 adverse event** | **2 (66.7)** | **0** | **4 (100)** | **2 (50.0)** | **14 (93.3)** | **2 (13.3)** | **10 (90.9)** | **4 (36.4)** | **1 (100)** | **1 (100)** | **4 (100)** | **2 (50.0)** | **35 (92.1)** | | **11 (28.9)** |
| Nausea | 2 (66.7) | 0 | 2 (50.0) | 1 (25.0) | 9 (60.0) | 0 | 8 (72.7) | 0 | 1 (100) | 0 | 4 (100) | 0 | 26 (68.4) | | 1 (2.6) |
| Diarrhoea | 1 (33.3) | 0 | 1 (25.0) | 0 | 4 (26.7) | 0 | 4 (36.4) | 0 | 1 (100) | 0 | 4 (100) | 0 | 15 (39.5) | | 0 |
| Vomiting | 0 | 0 | 2 (50.0) | 1 (25.0) | 5 (33.3) | 0 | 5 (45.5) | 0 | 0 | 0 | 0 | 0 | 12 (31.6) | | 1 (2.6) |
| Alanine aminotransferase increased | 0 | 0 | 0 | 0 | 3 (20.0) | 1 (6.7) | 3 (27.3) | 1 (9.1) | 0 | 0 | 1 (25.0) | 0 | 7 (18.4) | | 2 (5.3) |
| Hypotension | 0 | 0 | 0 | 0 | 1 (6.7) | 0 | 4 (36.4) | 3 (27.3) | 1 (100) | 1 (100) | 2 (50.0) | 1 (25.0) | 8 (21.1) | | 5 (13.2) |
| Fatigue | 0 | 0 | 0 | 0 | 7 (46.7) | 0 | 0 | 0 | 0 | 0 | 3 (75.0) | 0 | 10 (26.3) | | 0 |
| Asthenia | 0 | 0 | 1 (25.0) | 0 | 3 (20.0) | 0 | 2 (18.2) | 0 | 0 | 0 | 0 | 0 | 6 (15.8) | | 0 |
| Aspartate aminotransferase increased | 0 | 0 | 0 | 0 | 1 (6.7) | 1 (6.7) | 4 (36.4) | 1 (9.1) | 0 | 0 | 0 | 0 | 5 (13.2) | | 2 (5.3) |
| Dry skin | 0 | 0 | 0 | 0 | 1 (6.7) | 0 | 0 | 0 | 0 | 0 | 1 (25.0) | 0 | 2 (5.3) | | 0 |
| Rash | 0 | 0 | 0 | 0 | 3 (20.0) | 0 | 0 | 0 | 0 | 0 | 0 | 0 | 3 (7.9) | | 0 |
| Blood creatinine increased | 0 | 0 | 1 (25.0) | 0 | 1 (6.7) | 0 | 0 | 0 | 0 | 0 | 0 | 0 | 2 (5.3) | | 0 |
| Constipation | 1 (33.3) | 0 | 2 (50.0) | 0 | 1 (6.7) | 0 | 0 | 0 | 0 | 0 | 0 | 0 | 4 (10.5) | | 0 |

| **Preferred term, n (%)** | **LXS196 200 mg BID patients** | | **LXS196 300 mg BID (RDE) patients** | | **LXS196 400 mg BID patients** | | **All LXS196 BID patients** | | **All LXS196**  **patients** | |
| --- | --- | --- | --- | --- | --- | --- | --- | --- | --- | --- |
|  | **n=6** | | **n=18** | | **n=6** | | **n=30** | | **N=68** | |
|  | **All grades** | **Grade ≥3** | **All grades** | **Grade ≥3** | **All grades** | **Grade ≥3** | **All grades** | **Grade ≥3** | **All grades** | **Grade ≥3** |
| **Number of patients with ≥1 adverse event** | **5 (83.3)** | **0** | **17 (94.4)** | **2 (11.1)** | **6 (100)** | **4 (66.7)** | **28 (93.3)** | **6 (20.0)** | **63 (92.6)** | **17 (25.0)** |
| Nausea | 2 (33.3) | 0 | 14 (77.8) | 0 | 3 (50.0) | 0 | 19 (63.3) | 0 | 45 (66.2) | 1 (1.5) |
| Diarrhoea | 2 (33.3) | 0 | 11 (61.1) | 1 (5.6) | 3 (50.0) | 0 | 16 (53.3) | 1 (3.3) | 31 (45.6) | 1 (1.5) |
| Vomiting | 0 | 0 | 7 (38.9) | 0 | 2 (33.3) | 0 | 9 (30.0) | 0 | 21 (30.9) | 1 (1.5) |
| Alanine aminotransferase increased | 0 | 0 | 5 (27.8) | 1 (5.6) | 3 (50.0) | 1 (16.7) | 8 (26.7) | 2 (6.7) | 15 (22.1) | 4 (5.9) |
| Hypotension | 0 | 0 | 3 (16.7) | 0 | 4 (66.7) | 1 (16.7) | 7 (23.3) | 1 (3.3) | 15 (22.1) | 6 (8.8) |
| Fatigue | 0 | 0 | 3 (16.7) | 0 | 1 (16.7) | 0 | 4 (13.3) | 0 | 14 (20.6) | 0 |
| Asthenia | 1 (16.7) | 0 | 4 (22.2) | 0 | 2 (33.3) | 0 | 7 (23.3) | 0 | 13 (19.1) | 0 |
| Aspartate aminotransferase increased | 0 | 0 | 3 (16.7) | 1 (5.6) | 3 (50.0) | 1 (16.7) | 6 (20.0) | 2 (6.7) | 11 (16.2) | 4 (5.9) |
| Dry skin | 1 (16.7) | 0 | 4 (22.2) | 0 | 1 (16.7) | 0 | 6 (20.0) | 0 | 8 (11.8) | 0 |
| Rash | 0 | 0 | 4 (22.2) | 0 | 1 (16.7) | 0 | 5 (16.7) | 0 | 8 (11.8) | 0 |
| Blood creatinine increased | 3 (50.0) | 0 | 2 (11.1) | 0 | 0 | 0 | 5 (16.7) | 0 | 7 (10.3) | 0 |
| Constipation | 0 | 0 | 1 (5.6) | 0 | 2 (33.3) | 0 | 3 (10.0) | 0 | 7 (10.3) | 0 |

*All grade adverse events occurring in ≥10% “All LXS196 patients”.

A patient with multiple severity grades for an adverse event is only counted under the maximum grade. MedDRA version 24.1, CTCAE version 4.03.

BID, twice a day; CTCAE, Common Terminology Criteria for Adverse Events; MedDRA, Medical Dictionary for Regulatory Activities; QD, once daily; RDE, recommended dose for expansion.

**Supplementary Table 4: PK parameters**

|  | **Parameter** | **Statistics** | **LXS196** | | | | | | | | |
| --- | --- | --- | --- | --- | --- | --- | --- | --- | --- | --- | --- |
|  |  |  | **100 mg QD** | **200 mg QD** | **300 mg QD** | **500 mg QD** | **800 mg QD** | **1000 mg QD** | **200 mg BID** | **300 mg BID** | **400 mg BID** |
|  |  |  | **n=3** | **n=4** | **n=15** | **n=11** | **n=1** | **n=4** | **n=6** | **n=18** | **n=6** |
| **C1D1** | **AUC_inf_** | **n** | 2 | 3 | 15 | 10 | 1 | 3 | 6 | 16 | 5 |
|  | **(hr*ng/mL)** | **Mean (SD)** | 8640 (1660) | 21600 (4060) | 51300 (37600) | 63400 (21600) | 66200 (–) | 78000 (24500) | 29200 (23200) | 35200 (20800) | 30400 (12500) |
|  |  | **CV%** | 19.2 | 18.8 | 73.3 | 34.2 | – | 31.3 | 79.5 | 59.1 | 40.9 |
|  |  | **Geo-mean** | 8560 | 21300 | 42100 | 59500 | 66200 | 75700 | 24000 | 30900 | 28400 |
|  |  | **Geo-CV%** | 19.5 | 20 | 70.4 | 41.3 | – | 30.1 | 70.6 | 54.2 | 44.8 |
|  |  | **Median**  **(Min, max)** | 8640  (7470, 9810) | 22600  (17100, 25000) | 43100  (16000, 164000) | 61700  (24000, 103000) | 66200  (66200, 66200) | 66000  (61900, 106000) | 21600  (11900, 75000) | 28400  (13300, 83500) | 31700  (16500, 47800) |
|  | **AUC_0-t_** | **n** | 3 | 3 | 15 | 10 | 1 | 4 | 6 | 17 | 5 |
|  | **(hr*ng/mL)** | **Mean (SD)** | 8670 (2490) | 19100 (4270) | 40200 (26300) | 50700 (17000) | 54300 (–) | 74300 (30100) | 18600 (14500) | 20500 (10200) | 18100 (7690) |
|  |  | **CV%** | 28.7 | 22.4 | 65.4 | 33.5 | – | 40.6 | 77.8 | 49.7 | 42.4 |
|  |  | **Geo-mean** | 8440 | 18700 | 34000 | 48000 | 54300 | 69700 | 15400 | 18700 | 16800 |
|  |  | **Geo-CV%** | 28.5 | 22.9 | 64.6 | 38 | – | 43 | 69.5 | 45.5 | 46 |
|  |  | **Median**  **(Min, max)** | 8020  (6570, 11400) | 18900  (14900, 23400) | 34400  (13200, 118000) | 50700  (21400, 85500) | 54300  (54300, 54300) | 70100  (47000, 110000) | 13300  (7340, 47200) | 17000  (9150, 45800) | 18200  (9590, 29200) |
|  | **C_max_** | **n** | 3 | 3 | 15 | 10 | 1 | 4 | 6 | 17 | 5 |
|  | **(ng/mL)** | **Mean (SD)** | 776 (69.8) | 3540 (1250) | 5580 (3220) | 4970 (1800) | 6080 (–) | 5410 (2160) | 4070 (3390) | 4100 (1610) | 4350 (2530) |
|  |  | **CV%** | 9 | 35.2 | 57.7 | 36.3 | – | 39.9 | 83.3 | 39.3 | 58.3 |
|  |  | **Geo-mean** | 774 | 3410 | 4940 | 4670 | 6080 | 5080 | 3270 | 3850 | 3730 |
|  |  | **Geo-CV%** | 9.3 | 33.8 | 51.9 | 38.9 | – | 42.9 | 77.1 | 35.9 | 72.4 |
|  |  | **Median**  **(Min, max)** | 811  (696, 822) | 2860  (2780, 4980) | 5370  (2410, 15200) | 4380  (2420, 7740) | 6080  (6080, 6080) | 5230  (3260, 7920) | 2950  (1610, 10700) | 4210  (2420, 8860) | 3850  (1380, 8330) |
|  | **T_max_** | **n** | 3 | 3 | 15 | 10 | 1 | 4 | 6 | 17 | 5 |
|  | **(hour)** | **Median**  **(Min, max)** | 1  (0.500, 6.00) | 1  (0.500, 2.00) | 1  (0.483, 3.97) | 1.03  (0.500, 2.00) | 0.483  (0.483, 0.483) | 2  (1.00, 6.05) | 0.525  (0.483, 2.00) | 1  (0.333, 4.00) | 1.03  (0.467, 1.03) |
|  |  |  |  |  |  |  |  |  |  |  |  |
|  | **T_1/2_** | **n** | 2 | 3 | 15 | 10 | 1 | 3 | 6 | 16 | 5 |
|  | **(hour)** | **Mean (SD)** | 8.49 (1.41) | 8.52 (2.01) | 10.8 (1.65) | 10.2 (1.27) | 8.85 (–) | 10.0 (1.42) | 13.1 (1.76) | 12.1 (2.29) | 13.8 (2.94) |
|  |  | **CV%** | 16.6 | 23.6 | 15.3 | 12.5 | – | 14.2 | 13.5 | 18.9 | 21.4 |
|  |  | **Geo-mean** | 8.43 | 8.36 | 10.7 | 10.1 | 8.85 | 9.94 | 13 | 11.9 | 13.5 |
|  |  | **Geo-CV%** | 16.8 | 24.2 | 15.2 | 12.9 | – | 13.8 | 13.6 | 18.5 | 22.4 |
|  |  | **Median**  **(Min, max)** | 8.49  (7.49, 9.49) | 8.44  (6.56, 10.6) | 11.1  (7.92, 14.0) | 10.1  (7.73, 12.4) | 8.85  (8.85, 8.85) | 9.32  (9.06, 11.6) | 13.1  (10.5, 15.9) | 11.8  (8.76, 16.9) | 14.5  (10.2, 17.2) |

**Supplementary Table 4: PK parameters (continued)**

|  | **Parameter** | **Statistics** | **LXS196** | | | | | | | | |
| --- | --- | --- | --- | --- | --- | --- | --- | --- | --- | --- | --- |
|  |  |  | **100 mg QD** | **200 mg QD** | **300 mg QD** | **500 mg QD** | **800 mg QD** | **1000 mg QD** | **200 mg BID** | **300 mg BID** | **400 mg BID** |
|  |  |  | **n=3** | **n=4** | **n=15** | **n=11** | **n=1** | **n=4** | **n=6** | **n=18** | **n=6** |
| **C1D15** | **AUC_0-t_** | **n** | 2 | 3 | 13 | 9 | – | 2 | 5 | 12 | 4 |
|  | **(hr*ng/mL)** | **Mean (SD)** | 10400 (776) | 13200 (3260) | 30500 (18700) | 45700 (16700) | – | 48300 (7600) | 16900 (7530) | 19100 (5540) | 15900 (2810) |
|  |  | **CV%** | 7.4 | 24.7 | 61.3 | 36.6 | – | 15.7 | 44.6 | 29.1 | 17.7 |
|  |  | **Geo-mean** | 10400 | 12900 | 26500 | 43100 | – | 48000 | 15400 | 18300 | 15700 |
|  |  | **Geo-CV%** | 7.4 | 26.7 | 58.1 | 36.9 | – | 15.9 | 50.8 | 30.5 | 18.1 |
|  |  | **Median**  **(Min, max)** | 10400  (9900, 11000) | 13800  (9650, 16100) | 27900  (10800, 85000) | 41700  (26100, 71500) | – | 48300  (42900, 53600) | 16000  (8440, 25400) | 17400  (10100, 30300) | 16000  (12700, 18800) |
|  | **C_max_** | **n** | 3 | 3 | 14 | 9 | – | 2 | 5 | 17 | 4 |
|  | **(ng/mL)** | **Mean (SD)** | 1190 (217) | 2500 (252) | 3860 (2220) | 4170 (1930) | – | 5110 (2180) | 2910 (1990) | 2860 (569) | 2100 (462) |
|  |  | **CV%** | 18.2 | 10.1 | 57.6 | 46.2 | – | 42.6 | 68.6 | 19.9 | 22 |
|  |  | **Geo-mean** | 1180 | 2490 | 3500 | 3870 | – | 4870 | 2470 | 2800 | 2070 |
|  |  | **Geo-CV%** | 19.3 | 10.4 | 43.5 | 40.6 | – | 46.2 | 68.3 | 21.9 | 22.1 |
|  |  | **Median**  **(Min, max)** | 1260  (951, 1370) | 2620  (2210, 2670) | 3310  (2130, 10900) | 3700  (2280, 8830) | – | 5110  (3570, 6650) | 2540  (1250, 6280) | 2880  (1650, 4000) | 2060  (1620, 2680) |
|  | **T_max_** | **n** | 3 | 3 | 14 | 9 | – | 2 | 5 | 17 | 4 |
|  | **(hour)** | **Median**  **(Min, max)** | 1  (1.00, 4.00) | 1  (0.500, 1.00) | 1  (0.483, 2.05) | 1.07  (0.467, 2.00) | – | 0.792  (0.583, 1.00) | 1  (0.500, 4.00) | 0.567  (0.467, 4.00) | 0.842  (0.500, 1.95) |
|  | **C_min_** | **n** | 3 | 3 | 14 | 9 | – | 2 | 5 | 17 | 4 |
|  | **(ng/mL)** | **Mean (SD)** | 144 (58.0) | 143 (59.2) | 426 (319) | 660 (234) | – | 674 (10.6) | 787 (376) | 1040 (462) | 647 (194) |
|  |  | **CV%** | 40.3 | 41.4 | 75 | 35.4 | – | 1.6 | 47.7 | 44.5 | 30 |
|  |  | **Geo-mean** | 137 | 134 | 344 | 622 | – | 673 | 705 | 912 | 627 |
|  |  | **Geo-CV%** | 38.7 | 48.3 | 74.4 | 38.5 | – | 1.6 | 59.3 | 65.6 | 29.7 |
|  |  | **Median**  **(Min, max)** | 112  (109, 211) | 149  (81.1, 199) | 326  (122, 1330) | 643  (373, 1030) | – | 674  (666, 681) | 883  (368, 1150) | 1070  (155, 2100) | 608  (464, 909) |
|  | **R_acc_** | **n** | 2 | 2 | 13 | 8 | – | 2 | 5 | 12 | 3 |
|  |  | **Mean (SD)** | 1.27 (0.570) | 0.750 (0.145) | 0.934 (0.307) | 0.793 (0.214) | – | 0.718 (0.161) | 1.11 (0.611) | 1.15 (0.403) | 0.738 (0.204) |
|  |  | **CV%** | 44.9 | 19.3 | 32.8 | 26.9 | – | 22.4 | 54.9 | 35.1 | 27.7 |
|  |  | **Geo-mean** | 1.2 | 0.743 | 0.895 | 0.767 | – | 0.709 | 0.989 | 1.09 | 0.721 |
|  |  | **Geo-CV%** | 49.1 | 19.7 | 30.5 | 28.4 | – | 22.9 | 58.3 | 37.1 | 26.6 |
|  |  | **Median**  **(Min, max)** | 1.27  (0.867, 1.67) | 0.75  (0.647, 0.852) | 0.838  (0.577, 1.72) | 0.768  (0.484, 1.11) | – | 0.718  (0.604, 0.832) | 0.852  (0.539, 2.00) | 1.07  (0.549, 1.98) | 0.643  (0.599, 0.972) |

AUC_0-t_, area under the plasma-concentration time curve from timepoint 0 to time t; AUC_inf_, area under the plasma concentration-time curve from time 0 to infinity; BID, twice a day; C1D1, cycle 1 day 1; C1D15, cycle 1 day 15; C_max_, maximum concentration; C_min_, trough concentration; CV, coefficient of variation; QD, once daily; R_acc_, accumulation ratio; SD, standard deviation; T_1/2_, terminal elimination half-life; T_max_, time to reach maximum plasma concentration.

**Supplementary Table 5: Mutation frequency A) of *GNAQ* and *GNA11*; and B) *BAP1* alleles**

**A) Mutation frequency of *GNAQ* and *GNA11***

| ***GNA11* (*GNA11* mutation)** | ***GNAQ* (*GNAQ* mutation)** | | | | |
| --- | --- | --- | --- | --- | --- |
| **Frequency** | ***GNAQ_Q209L*** | ***GNAQ_Q209P*** | ***GNAQ_R183Q*** | **Not detected** | **Total** |
| ***GNA11_Q209L*** | 0 | 0 | 0 | 22 | 22 |
| ***GNA11_R183C*** | 0 | 0 | 0 | 6 | 6 |
| **Not detected** | 2 | 13 | 1 | 4 | 20 |
| **Total** | 2 | 13 | 1 | 32 | 48 |

**B) Mutation frequency *BAP1* alleles**

| ***BAP1* mutation** | | | | |
| --- | --- | --- | --- | --- |
| ***BAP1*** | **Frequency** | **Percent** | **Cumulative frequency** | **Cumulative percent** |
| ***BAP1_AR145*** | 1 | 2.08 | 1 | 2.08 |
| ***BAP1_E166**** | 1 | 2.08 | 2 | 4.17 |
| ***BAP1_E642*** | 1 | 2.08 | 3 | 6.25 |
| ***BAP1_ESKSASNKSPL*** | 1 | 2.08 | 4 | 8.33 |
| ***BAP1_G554*** | 1 | 2.08 | 5 | 10.42 |
| ***BAP1_GYAIGNA128G*** | 1 | 2.08 | 6 | 12.50 |
| ***BAP1_H169Y*** | 1 | 2.08 | 7 | 14.58 |
| ***BAP1_L97R*** | 2 | 4.17 | 9 | 18.75 |
| ***BAP1_LAKAHNSHAR1*** | 1 | 2.08 | 10 | 20.83 |
| ***BAP1_NGLSAVRTMEA*** | 1 | 2.08 | 11 | 22.92 |
| ***BAP1_Q28P?*** | 1 | 2.08 | 12 | 25.00 |
| ***BAP1_Q665**** | 1 | 2.08 | 13 | 27.08 |
| ***BAP1_R179W*** | 1 | 2.08 | 14 | 29.17 |
| ***BAP1_R60**** | 1 | 2.08 | 15 | 31.25 |
| ***BAP1_RRS56*** | 1 | 2.08 | 16 | 33.33 |
| ***BAP1_S90*** | 1 | 2.08 | 17 | 35.42 |
| ***BAP1_VSTL62*** | 1 | 2.08 | 18 | 37.50 |
| ***BAP1_W52**** | 2 | 4.17 | 20 | 41.67 |
| ***BAP1_Y33**** | 1 | 2.08 | 21 | 43.75 |
| **Not detected** | 27 | 56.25 | 48 | 100.00 |

**Supplementary Figure 1: Dose levels and schedules tested in the dose escalation part to determine RDE**


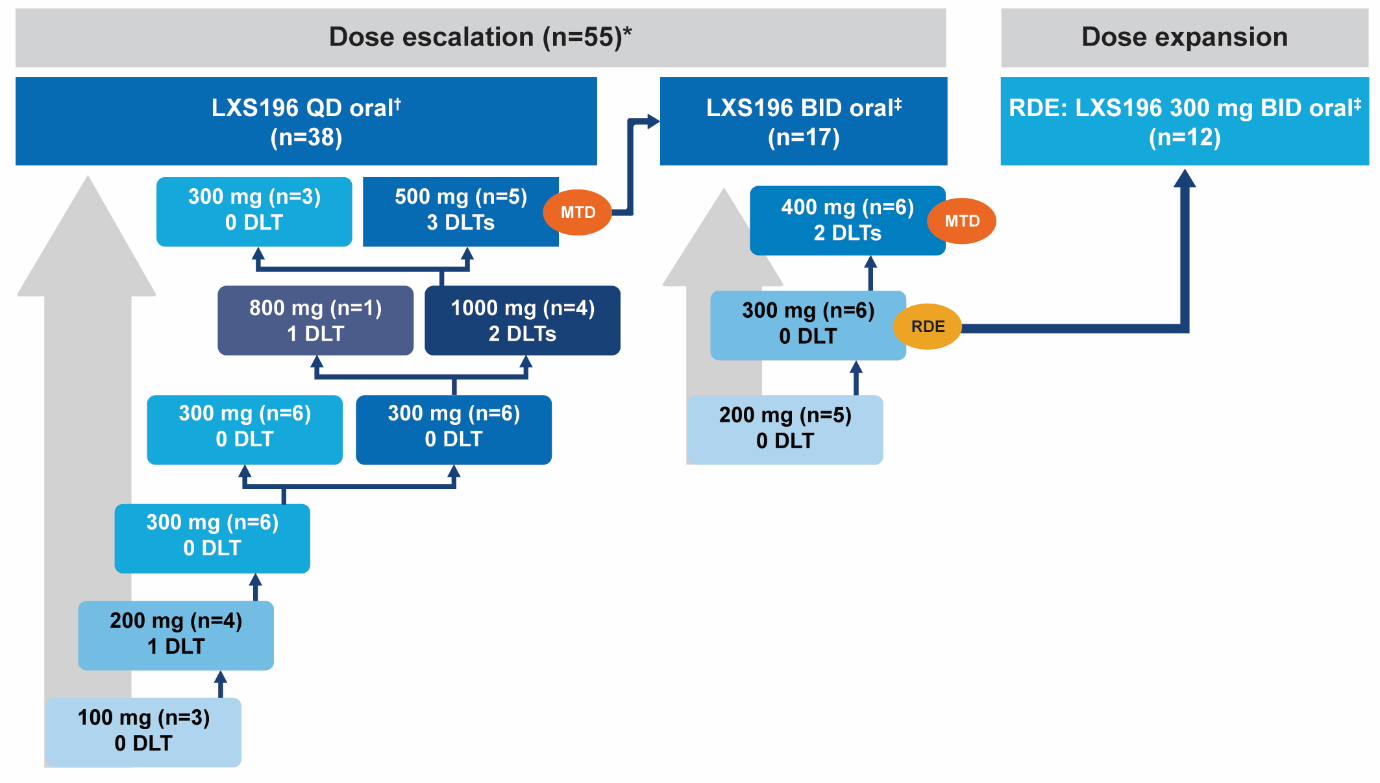


BID, twice daily; DLT, dose limiting toxicity; MTD, maximum tolerated dose; n, number of patients evaluable for dose determination per cohort; QD, once daily, RDE, recommended dose for expansion.

*Total 56 patients were enrolled and treated; one patient excluded from the analysis due lack of sufficient exposure to study treatment during cycle 1. **^†^**Dose QD except for C1D2 when no dose was administered. ^‡^Dosed BID except for C1D1 when only the morning dose was administered and C1D2 when no doses were administered.

DLTs are presented in Table 2A.

**Supplementary Figure 2: Gene expression levels from RNAseq patient samples**


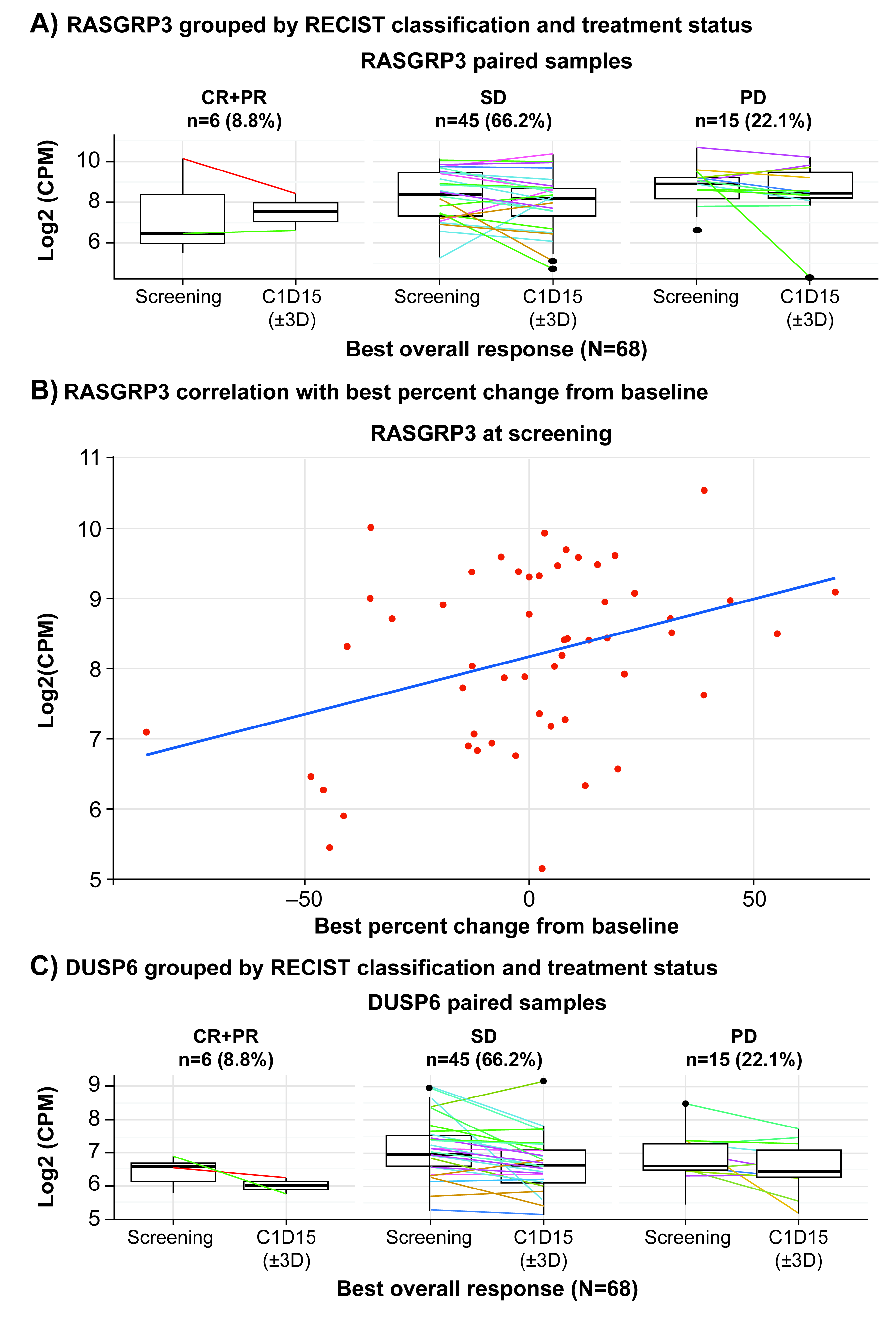


C1D15, cycle 1 day 15; D, day; CPM, counts per million; PD, progressive disease; PR, partial response; RECIST, Response Evaluation Criteria in Solid Tumours; SD, stable disease.
